# Synthesis and characterization of partially silane-terminated polyurethanes reinforced with acid-treated halloysite nanotubes for transparent armour systems

**DOI:** 10.1038/s41598-020-70661-3

**Published:** 2020-08-14

**Authors:** Rafaela Aguiar, Ronald E. Miller, Oren E. Petel

**Affiliations:** Department of Mechanical and Aerospace Engineering, Carleton University, Ottawa, ON K1S 5B6 Canada

**Keywords:** Composites, Polymers, Structural properties, Mechanical properties, Synthesis and processing

## Abstract

In the present work, nanocomposites based on the partially silane-terminated polyurethanes reinforced with sulfuric acid-treated halloysite nanotubes were synthesized and evaluated as a potential candidate for transparent blast resistant configurations. The polyurethane must present high tensile ductility at high strain rates to be able to contain fragments and increase the survivability of the system. Gas-gun spall experiments were conducted to measure the dynamic tensile strength (spall strength) and fracture toughness of the nanocomposite and neat polyurethane. The nanocomposite presented a 35% higher spall strength and 21% higher fracture toughness compared to the neat polyurethane while maintaining transparency. The recovered samples following the spall tests were analysed via scanning electron microscope fractographies. The nanocomposite and neat polyurethane samples were chemically characterized via Fourier transform infrared spectroscopy and melting behaviour via differential scanning calorimetry. The improved properties can be attributed, in large part, to the presence of more rigid spherulitic structures, and a rougher fracture surface constituting of several micro-cracks within the nanocomposite.

## Introduction

Transparent armour systems must protect against blast and ballistic threats while maintaining structural integrity and optical transparency. Generally, transparent armour systems are composed of laminated glasses sheets bonded together by thin adhesive interlayers of polyvinyl butyral, polyurethane, and/or ethylene–vinyl acetate films, normally combined with polycarbonate as a backing layer. To achieve ballistic protection requirements; the glass layers are generally much thicker than the polymeric layers, which leads to thick and heavy armour solutions^1,2^. Material improvements that allow weight reductions among transparent armour systems are of great interest for personal and vehicular applications.

**Figure 1:**
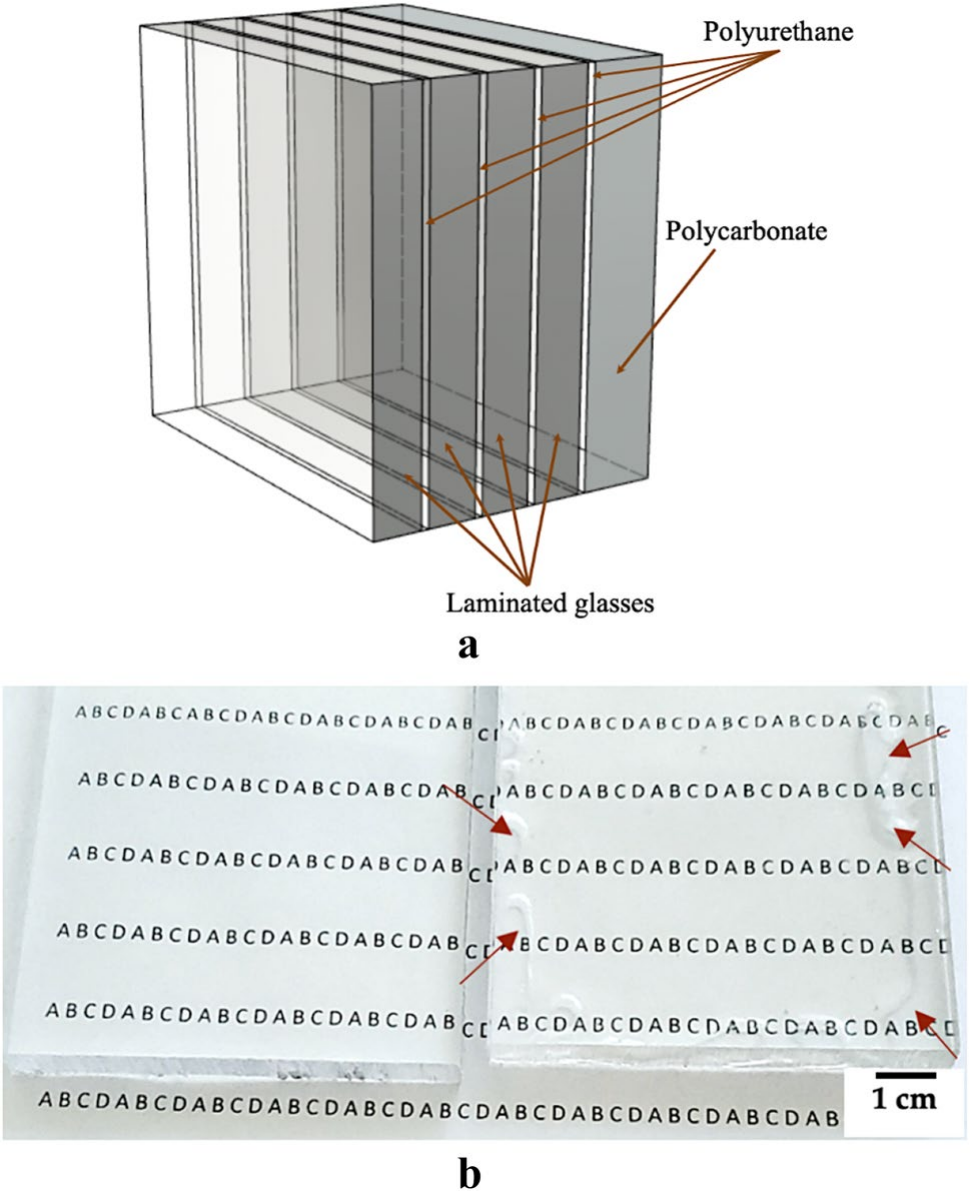
(**a**) Illustrative representation of a typical transparent armour configuration and (**b**) transparency evidence of silane terminated PU reinforced with HNT. A 9.5-mm-thick polycarbonate plate without a backing layer (left) and the same plate backed with a 1.5-mm layer of the nanocomposite (right).

Polymers are extensively used in armour applications. Stretched polymers fibers (e.g., Aramids) are widely used in the ballistic fabrics integrated into soft armour and spall liner applications^3^. Transparent polymers have historically seen broad use in transparent armour applications, and while they continue to be used as cost-effective solutions in some visor and ballistic eyewear applications, their primary role in more robust armour solutions has been relegated to interlayer or backing support for transparent ceramics^1,4^. Figure 1a shows an illustrative representation of a typical transparent armour structure. Polyurethanes (PU) are commonly adopted as interlayers in ceramic laminated systems due to their high-tensile ductility and adhesive properties, which provides the containment of armour fragments and increases the spall resistance of the ceramic layers^5^. Figure 1b presents a relative transparency comparison between a 9.5-mm-thick polycarbonate plate with and without a halloysite/polyurethane nanocomposite adhesive backing layer. The adhesive layer consisted of a 1.5-mm-thick layer of silane-terminated PU reinforced with HNT. The red arrows show the edges of the backing layer, beyond which the layer is thin or not evenly applied, however, the central region of the two plates can be compared for their relative transparency.

Polyurethanes can be defined as a class of polymers which have urethane group in their structure. Polyurethanes are block copolymers, formed by a combination of hard and soft domains. The mechanical properties of PUs are related to the relative volumetric fractions of these hard and soft segments, the intrinsic properties of each block, the details of molecular packing of the constituents within the phases, and the density of hydrogen bonding. From a macromolecular scale perspective, the structure of PU consists of a soft matrix with hard domains acting as a reinforcement segmented phase^6^.

Halloysite (HNT) is a hydrated polymorph of the kaolin group, which includes kaolinite, dickite and nacrite. Kaolin minerals are 1:1 dioctahedral clays, with the empirical formula ${\text{Al}}_{2} {\text{Si}}_{2} {\text{O}}_{5} \left( {{\text{OH}}} \right)_{4}$. The external surface of the HNT has a tetrahedral sheet structure that consists of siloxane groups (Si–O–Si), while its internal structure has a gibbsite octahedral structure is formed by aluminol groups (Al–OH). The hydrated HNT presents a basal spacing (d_001_) of 10 Å, and the dehydrated HNT has a 7 Å spacing^7,8^. Kaolinite and HNT can frequently be found together. The kaolinite generally presents a platy morphology, while the HNTs can exhibit tubular, spheroidal, or platy morphologies^9^. Information about the mesoscopic structures of HNTs obtained from different geological deposits can be obtained via Small-Angle Neutron Scattering^10^. The characteristic dimensions of HNTs varying over ranges of 300–1,500 nm in length, 40–120 nm for the outer diameter, and 15–100 nm for the inner diameter. Some of the main characteristics of this aluminosilicate are high aspect (L/D) ratio, high mechanical strength and modulus, the possibility of modifying its polar surface, straight morphology with no entanglement, low cost, and availability in abundance^8^.

Published results indicate that the introduction of a low content of HNT can substantially improve mechanical and thermal properties of PU based materials. Gong et al.^11^ reported an increase of 35% in tensile strength of NCO-terminated castor oil-based PU with the introduction of 0.5 wt% of HNTs. Wu et al.^12^ synthesized waterborne PU reinforced with aminosilane modified HNT for coating applications and, reported an increase in 200% in tensile strength and 30% increase in elongation at break. Mohammadzadeh et al.^13^ reported the increase in phase separation of shape-memory thermoplastic PU due to the incorporation of 1 and 2 wt% HNT. They described an increase in the crystallinity of the polymeric matrix due to a nucleation effect of the HNT in the crystallization process^13^. Smith et al.^14^ reduced the flammability of PU foam by introducing multilayer nanocoatings based on HNT stabilized by poly (acrylic acid) or by branched polyehylenimine, deposited via aqueous suspensions. Cone calorimetry results indicated a reduction of 61.7% in the peak heat release rate and a 60.1% decrease in the total smoke release.

The reactivity of the HNTs is limited to Si–OH and Al–OH groups that are exposed due to HNT surface and crystallographic defects^15^. The treatment of HNT with sulfuric acid can increase its reactivity, through a reaction between the acid and both the inner and outer surfaces of the nanotubes. Thus, the density of potential sites for bonding increases through the breakage of the HNT structures via dissolution of the AlO_6_ octahedral layers and the breakdown and collapse of SiO_4_ tetrahedral layers^16^. As a consequence of this reactivity increase, the dispersion of HNTs can be improved within the polymeric matrix, improving the overall transparency and mechanical properties of the nanocomposite^17^.

The introduction of inorganic groups in an organic polymeric structure can generate hybrid materials with outstanding properties, that have different potential applications, which depend on their building block combinations^18^. The addition of silane groups even in small quantities can improve different material properties in polymeric materials^19^. Luca et al.^20^ synthesised hybrid films from epoxidised castor oil, γ-glycidoxypropyltrimethoxysilane and tetraethoxysilane that had high adhesion to aluminum surfaces, with increased hardness and tensile strength, which increased with the concentration of inorganic precursors. Wang et al.^21^ developed waterborne PU/nanosilica composites with triethoxysilane side chain groups. These composites resulted in an increase to the tensile strength and hardness at low concentrations, but these properties decreased with increasing concentrations of nanosilica incorporation. The authors proposed that this response was due to the anchoring of these nanosilica particles onto the side chain of the PU due the condensation reaction between the surface silanol groups of nanosilica and the triethoxysilane groups in the side chains of PU. Wu et al.^22^ overcame the very high incompatibility between waterborne PU and polysiloxane via the introduction of alkoxysilane groups in waterborne PU’s prepolymer. The authors proved the good dispersibility of the polysiloxane via transmission electron microscopy and dynamic light scattering analysis.

In this work, we developed a PU/HNT nanocomposite that maintains high transparency, while significantly improving both its dynamic tensile strength and fracture toughness in comparison to the neat PU polymer. The PU’s prepolymer was partially terminated through a reaction between the NCO terminations of the prepolymer and a secondary aminosilane (3-(N-ethylamino) isobutyl) trimethoxysilane). This reaction resulted in monodentate urea linkages and trimethoxysilane terminations in the PU’s prepolymer^23^. The hydrolysis of these silane terminations during the post-curing can possibly result in silanol terminations in the prepolymer end groups, which would potentially react via condensation with surface hydroxyl groups of HNT^16^. The secondary aminosilane was added at a weight fraction of 0.6% of the pre-polymer weight. A lower content was preferred to prevent a significant increase in the viscosity of the HNT/prepolymer solution. It should be noted that the same curative was selected for the neat PU and nanocomposite.

As PU plays an important role in providing tensile ductility to an armour system, the tensile behaviour under high strain rates was evaluated in terms of its dynamic tensile (spall) strength and fracture toughness in a classic spall failure test configuration. Post-spall recovery of the impacted polymers enabled an evaluation via Scanning Electronic Microscopy of alterations to the fracture surface of the polymer.

**Figure 2:**
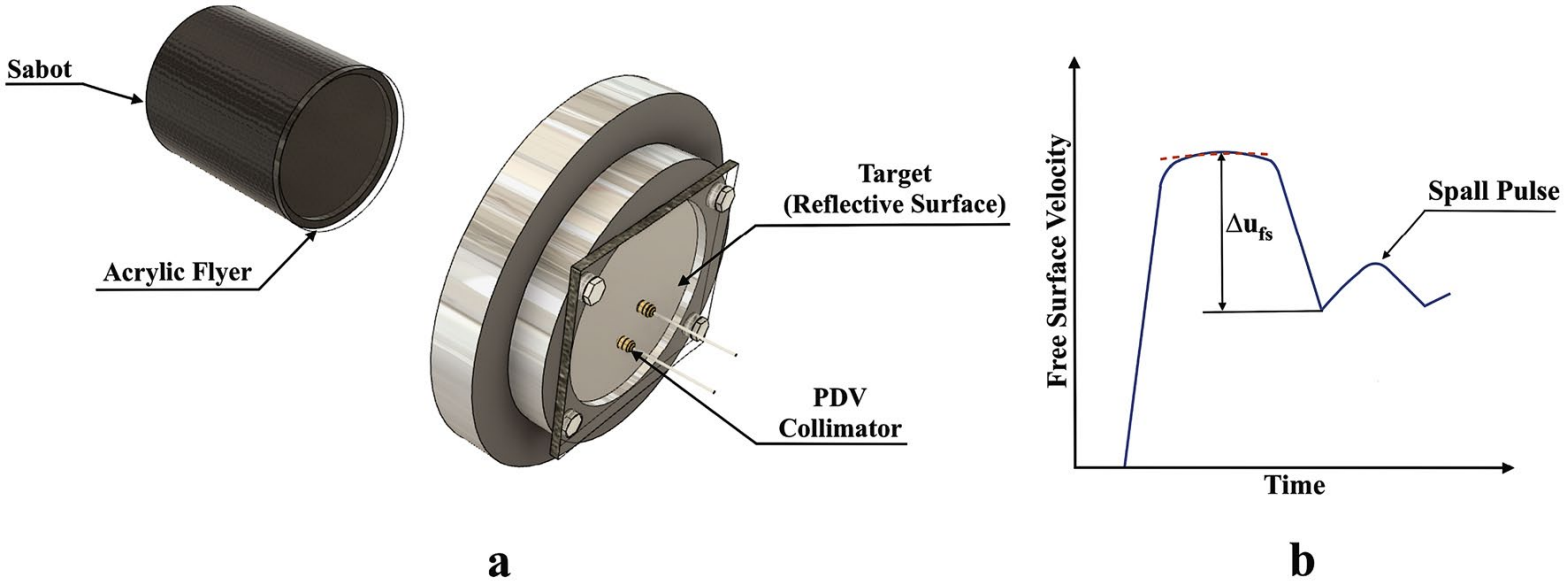
(**a**) Schematic configuration of the gas-gun setup used on the Spall Testing and (**b**) Illustrative free surface velocity history of a spalled material.

The spall testing was carried out in a 64-mm smooth-bore single-stage light gas gun at the Impact Research Lab facility at Carleton University. These experiments were conducted to measure the dynamic tensile strength and fracture toughness of the polymeric samples. Acrylic flyer plates were selected to induce spall in the nanocomposite and neat PU, due to the requirement for plate rigidity and the similarity of their shock Hugoniots^24^. The back-face of the samples were coated with Silver using the SC7620 Quorum sputter coater to provide a reflective surface. The velocity histories of the free surface were measured using a two-channel photonic Doppler velocimeter (PDV)^25,26^. A schematic of the experimental configuration is illustrated in Fig. 2a.

The analysis of the free surface velocity history of the spalled sample (Fig. 2b) provides insight into the fracture stress and failure kinetics^27^. After the impact, compression waves propagate in opposite directions in the flyer and target materials introducing a steep rise in the free-surface velocity and saturation to a value proportional to the impact velocity. When these waves reach the free surface, they are reflected as rarefaction fans leading to a progressive decrease on the free surface velocity. The slope of this velocity decay is proportional to the tensile strain rate in the target. The spallation occurs if the resultant tensile stresses are high enough to generate macroscopic failure by material separation. A compressive disturbance (Spall Pulse) is generated by the relaxation of the tensile stress at fracture, resulting in an increase of the back-face velocity^24,28^. Information about fracture kinetics can be obtained from the analysis of the flow associated with stress relaxation during spalling^29^.

The spall strength (σ_sp_) was calculated using a linear approximation that accounts for elastic–plastic effects using the acoustics approach provided by Stepanov^30^

1 $$ {\upsigma }_{{{\text{sp}}}} = {\rho C}_{{\text{L}}} \Delta {\text{u}}_{{{\text{fs}}}} \frac{1}{{1 + { }\frac{{{\text{C}}_{{\text{L}}} }}{{{\text{C}}_{0} }}}}, $$

where $\rho$, ${\text{C}}_{{\text{L}}}$, C_0_ and $\Delta {\text{u}}_{{{\text{fs}}}}$ represent the initial density, longitudinal sound speed, bulk sound speed, and pullback velocity of the free surface, respectively. The acoustic properties of the polymers were determined using an Olympus 45MG ultrasonic thickness gage coupled with a delay line transducer at a frequency of 10 MHz. The sound speeds were measured to be 1.98 km/s and 2.03 km/s for the neat PU and nanocomposite, respectively. The strain rate under tensile unloading ($\dot{\varepsilon }_{{\text{u}}}$) was calculated using^31^.

2 $$ \dot{\varepsilon }_{{\text{u}}} = \frac{1}{{2{\text{C}}_{0} }}\left. {\frac{{{\text{du}}_{{{\text{fs}}}} \left( {\text{t}} \right)}}{{{\text{dt}}}}} \right|_{{{\text{unloading}}}} , $$

The strain rate magnitudes experienced by the samples during tensile unloading are on the magnitude of 10^4^ s^−1^, thus, the PU presents a glassy-like behaviour under these conditions and the PU failure occurs in a brittle fashion^32^. The fracture toughness (K_C_) was calculated using the equation proposed by Grady^33^ for brittle solids

3 $$ K_{c} = \sqrt {\frac{{\sigma_{sp}^{3} }}{{3\rho_{0} C_{0} \dot{\varepsilon }_{{\text{u}}} }}} . $$

## Results and discussion

**Figure 3:**
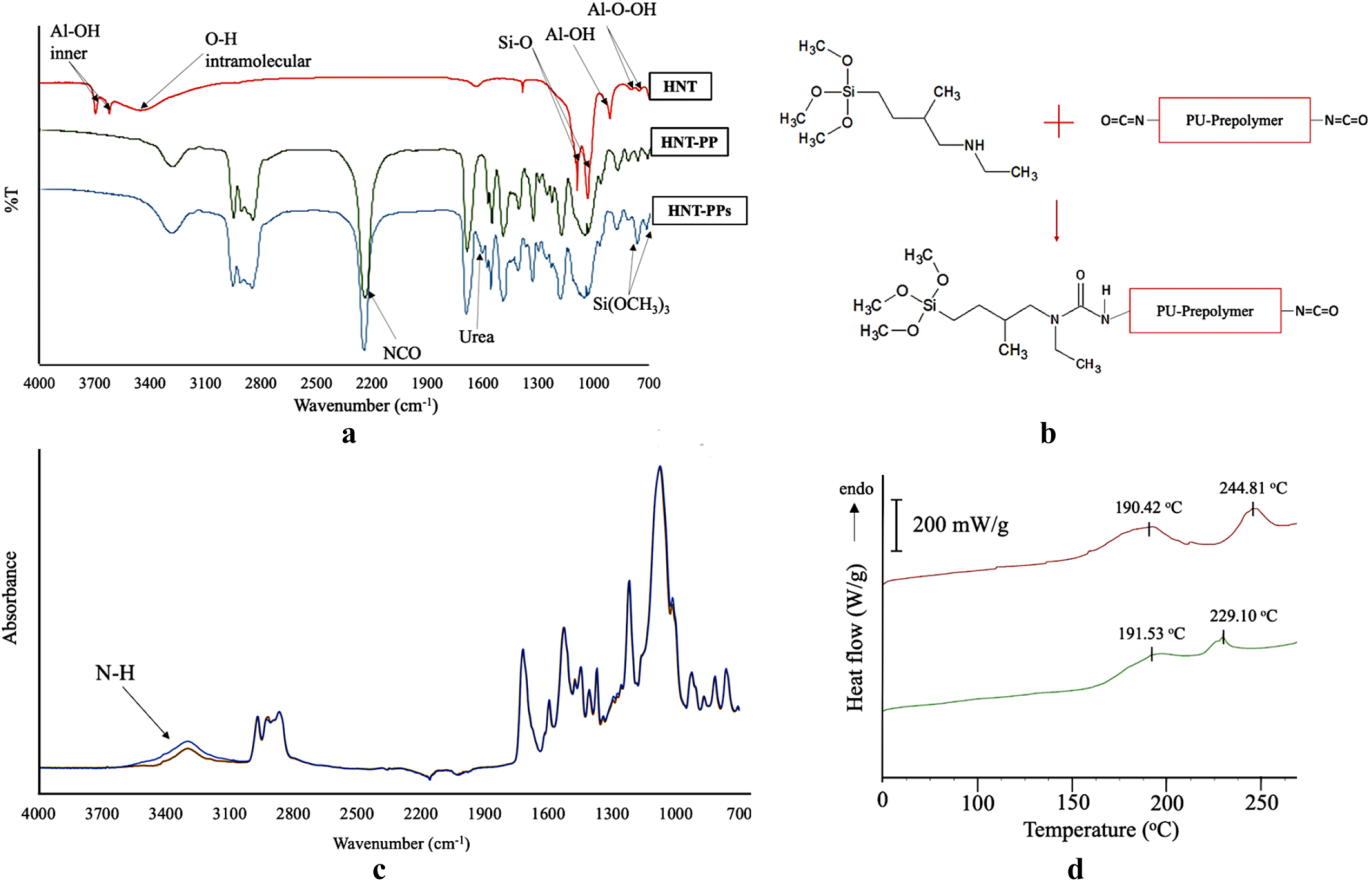
(**a**) FTIR spectra (green) of prepolymer with dispersed HNT (HNT-PP), and (blue) HNT-PP after addition of aminosilane; (**b**) scheme of the reaction between the PP and aminosilane; (**c**) FTIR spectra (green) of cured neat PU and (blue) nanocomposite and (**d**) DSC curves of (green) neat PU and (maroon) HNT-PU nanocomposite.

The Fourier transform infrared spectroscopy (FTIR) spectra for the acid-treated HNT is presented on Fig. 3a, where we observed the O–H stretching of the inner surface Al–OH at 3,695 cm^−1^ groups and inner Al–OH groups at 3,620 cm^−1^. The inner O–H deformation vibration was detected at 910 cm^−1^^16^. The inner Si–O stretching vibration was detected at 1,033 and 1,089 cm^−1^ and intramolecular O–H at 3,427 cm^−1^^34^. The FTIR spectra for PU prepolymer with dispersed HNT before and after the incorporation of the secondary aminosilane are shown in Fig. 3a. The reaction was monitored based on the intensity decrease of the NCO peak (2,270 cm^−1^) and emergence of urea (C=O) peaks at 1,610 cm^−1^, hence the reaction between the prepolymer’s NCO groups and secondary amine results in urea linkages (Fig. 3b). The resulting trimethoxysilane terminations can be observed at 817–774 cm^−1^. The reaction was observed to reach completion in approximately 8 min.

Considering the measured FTIR spectra of the neat PU and the HNT-PU (Fig. 3c) nanocomposite after cure, we observed the disappearance of the NCO peak for both materials, indicating the completion of the cure. The nanocomposite presented a more intense and broader N–H stretching peak. This peak shifts from 3,297 cm^−1^ for the neat PU to 3,302 cm^−1^ for the nanocomposite. The upward shift of this absorption band likely occurs due to an increase in the overall hydrogen bonding density^35^.

Through DSC results of the neat-PU and the nanocomposite (Fig. 3d) we identified two endothermic peaks at elevated temperatures. These peaks are related with different morphologies of hard segments crystallites; the lower temperature peak (Type I) corresponding to the melting of more phase mixed (lower rigidity) crystallites and, the higher temperature (Type II) associated with high order (tightly packed) crystallites^36^.

Both samples presented very similar values of melting temperature for Type I crystallites around 190 °C, however the HNT-PU material presented a Type II peak at 244.81 °C and the neat-PU at 229.10 °C. The higher melting point of HNT-PU’s Type II crystallites is likely due to a higher density of hydrogen bonding of N–H groups from the hard domain segments. This is evidence of a more phase-segmented structure in the HNT-PU nanocomposite.

**Figure 4:**
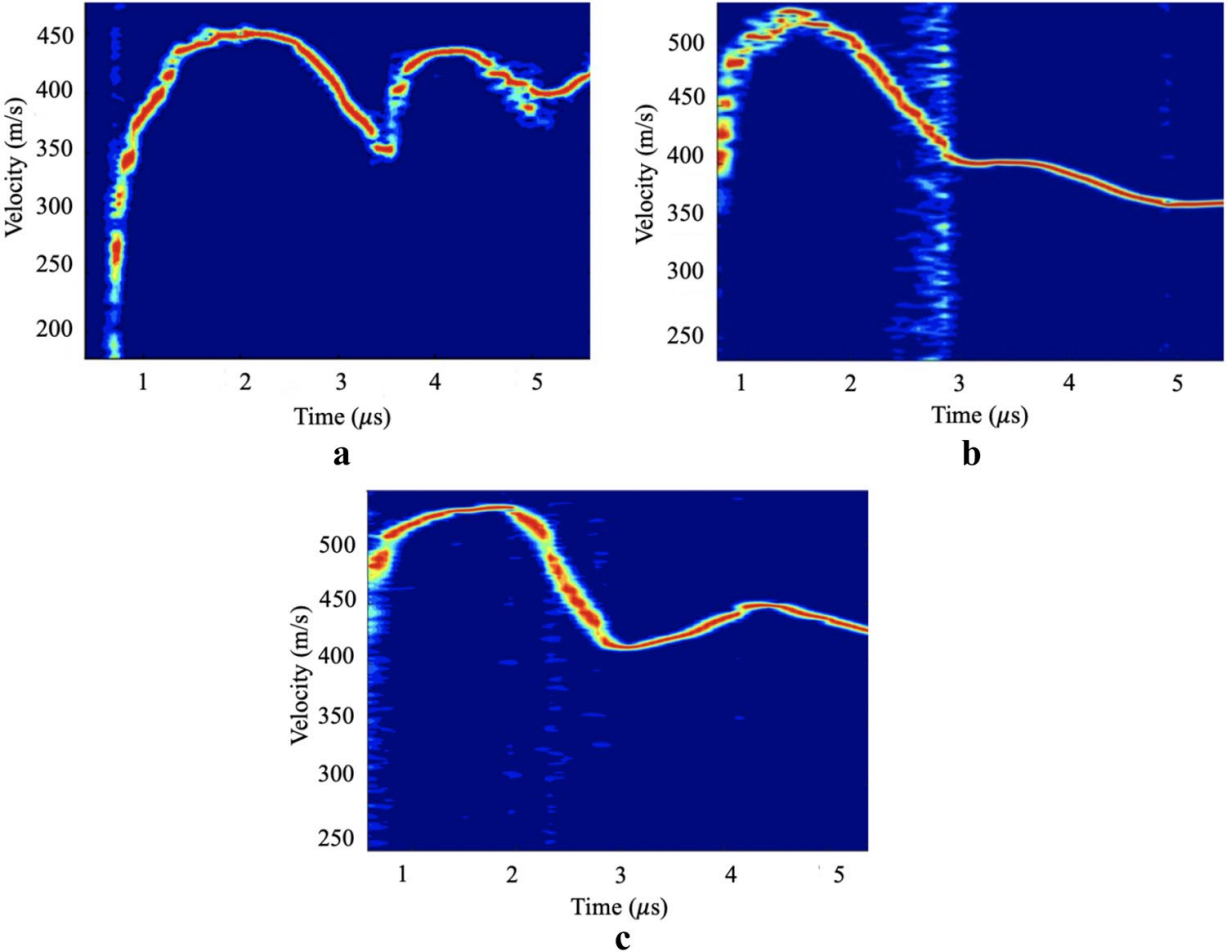
Velocity histories for (**a**) neat PU; (**b**) HNT-PU and (**c**) HNT-PU without silane end-groups at the same strain rate during tensile unloading.

Spectrogram profiles of the flyer plate-impacted samples (Fig. 4) based on the back-face velocity history were used to determine information about spall strength for a fixed tensile strain-rate. The results showed a strain rate during tensile unloading of approximately 2.75 (10^4^) s^−1^ for the neat PU and 2.76 (10^4^) s^−1^ for the nanocomposite. The spall strengths of the neat PU and, the nanocomposite were found to be 105 MPa and 143 MPa, respectively. The spall pulse from the neat PU velocity profile (Fig. 4a) presents a very sharp front, which indicates a high rate of fracture at the spall plane. This is in stark contrast with the spall pulse in the nanocomposite (Fig. 4b), at similar strain rate, where the slow and weak spall pulse response indicates a decrease in the damage evolution rate. The differences in pulse shape is further evidence of a favorable material response in the nanocomposite, which fails through a more energy dissipative failure mechanism. The back-face velocity history of the nanocomposite without silane end-groups can be seen on Fig. 4c. For this composite, the measured spall strength was 129 MPa, which was measured for a tensile unloading strain rate of approximately 2.79 (10^4^) s^−1^. Considering the spall values obtained for the HNT-PU and the neat PU, the spall strength of the HNT-PU composite without silane end-groups was determined to have an intermediate value of spall strength at the same strain rates.

**Figure 5:**
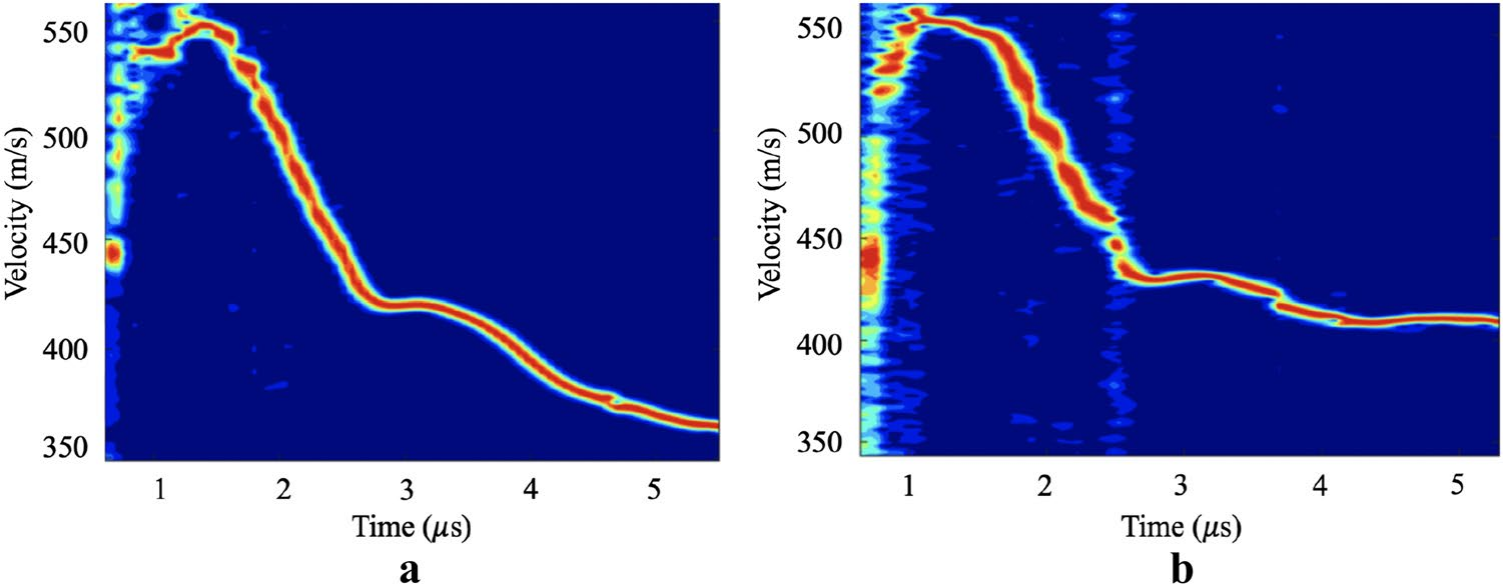
Velocity histories for (**a**) neat PU and (**b**) HNT-PU nanocomposite at the same dynamic loading conditions.

The fracture toughnesses were measured from spall tests that had similar peak shock stresses of 0.96 GPa for the neat PU and 0.99 GPa for the nanocomposite. Based on their free-surface spectrogram profiles (Fig. 5), the neat PU presented a spall strength of 134 MPa at a strain rate of 3.16 (10^4^) s^−1^ and HNT-PU material spall strength of 149 MPa at a strain rate of 2.83 (10^4^) s^−1^. The fracture toughnesses were found to be 3.41 and 4.13 MPa m^1/2^ for the neat PU and nanocomposite, respectively. These results show that the nanocomposite have a 35% higher spall strength and 21% higher fracture toughness compared to the neat PU under similar dynamic conditions.

**Figure 6:**
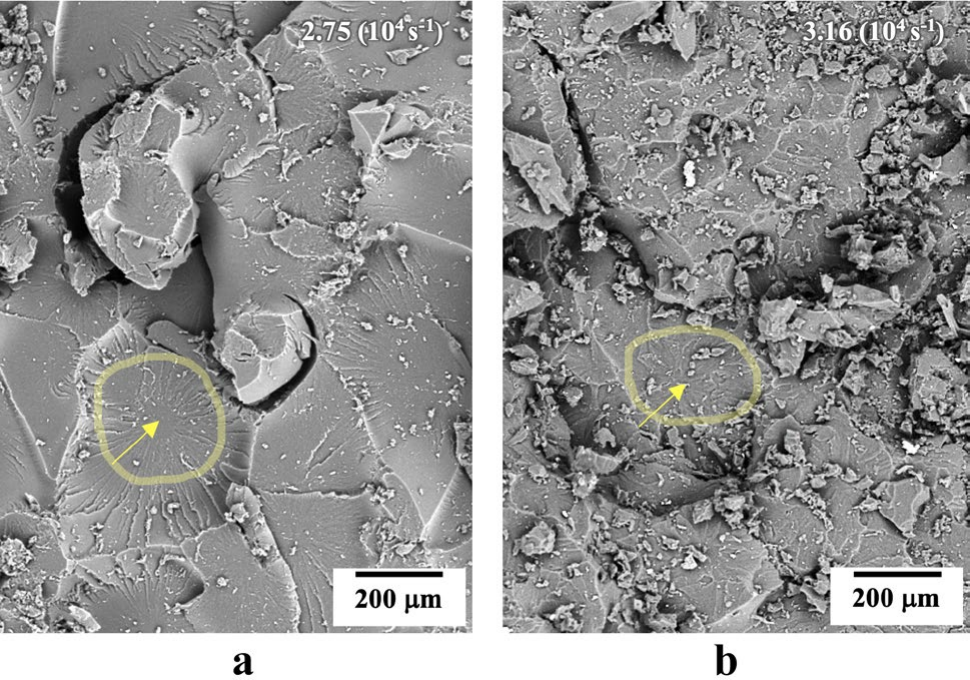
SEM images of fractured spalled surface of neat PU showing spherulitic morphology at tensile strain-rates of (**a**) 2.75 (10^4^) s^−1^ and (**b**) 3.16 (10^4^) s^−1^.

Rate-dependent behaviour was observed for all samples, as the spall strength increased with the tensile strain rate. This can be observed by comparing the velocity histories of the neat PU in Figs. 4a and 5a, where with the increase of tensile strain-rate a decrease on the slope of the spall pulse front occurs, again indicates a decrease in the damage evolution rate, evidence of greater energy dissipation in the PU failure mode^29^. Through the analysis of the SEM fractography of the samples spalled surfaces (Fig. 6), the neat PU at a higher tensile strain rate presented a rougher fracture surface with several micro-cracks. The presence of spherulitic superstructures (spherical semi-crystalline regions) can be noticed on the fracture surface of the neat PU (indicated on Fig. 6), where we also see a tendency for cracks to propagation around the spherulitic regions.

**Figure 7:**
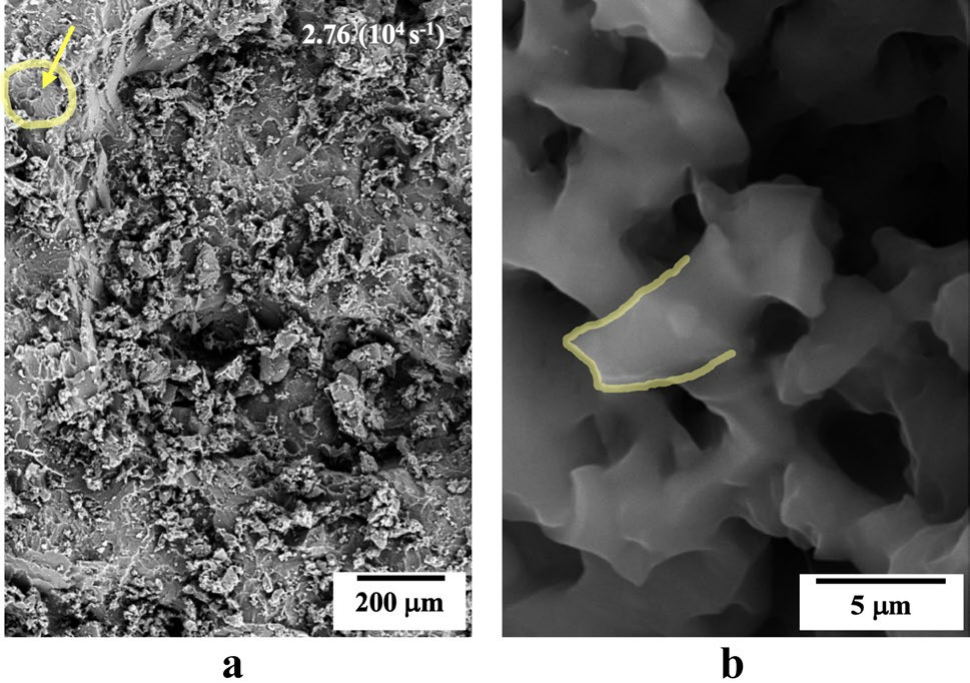
SEM image of fractured spalled surface HNT-PU nanocomposite at strain-rate of 2.76 (10^4^ s^−1^) (**a**) rough fractured surface with highlighted spherulitic structure; (**b**) evidence of heavily-coated nanotube.

Comparing the fractographies of the neat PU (Fig. 6a) and the nanocomposite (Fig. 7a) for the same tensile strain rate, the nanocomposite presented a rougher fracture surface with several micro-cracks. This evidence suggests that the crack propagation in the nanocomposite occurred at a lower strain rate than the comparative neat PU sample^37^. From Fig. 7a, we observed well-dispersed and heavily-coated nanotubes within the nanocomposite (Fig. 7a), which indicates good interfacial adhesion between the filler and the PU matrix. The incorporation of these HNTs influences the micro-crack nucleation mechanism involved in the spall process in the PU and was also seen qualitatively to influence the spherulitic size of the nanocomposite (Fig. 7b). This suggests an interference of the HNTs on the spherulite nucleation process within PU. The more tortuous crack propagation path due to the mechanisms of multiple micro-cracks and spherulitic deviation are possibly the main toughening mechanisms of the nanocomposite^38^.

This work shows how the partial amino-silane end-capping of PU pre-polymer and incorporation of 0.8wt% of HNT can significantly improve the dynamic response of the PU while maintaining transparency as a thin layer. The nanocomposite presented a 35% higher spall strength and 21% higher fracture toughness compared to the neat PU under the studied dynamic conditions.

FTIR and DSC results provide evidence of enhanced rigidity in the crystallite structures of the nanocomposite. Furthermore, through the analysis of the SEM fractography of the spalled surfaces, the nanocomposites presented a fracture mechanism with higher energy dissipation than the neat PU. The reinforcement in the macromolecular structure, combined with the ability of the HNTs to act as multiple sites for micro-crack nucleation are possibly the main toughening mechanisms of the nanocomposite.

The obtained results present the potential of amino-silane end-capping in PU prepolymer formulations as a way to improve chemical compatibility between filler and matrix. The composite material formulation strategy, which was focused on the ability of the HNTs to favorably alter the polymeric macromolecular structure during polymerization rather than the idea of the HNTs as a reinforcing fiber in a traditional composite, has the potential to improve other polymer-based composite systems.

## Materials and methods

### Materials

Silane-terminated PU was produced through a reaction between the prepolymer (Poly (propylene glycol), tolylene 2,4) with NCO content of 7.4% was obtained from Taiwan PU corporation and (3-(N-ethylamino) isobutyl) trimethoxysilane purchased from Gelest Inc. The curative used was 4,4′-Methylenebis (2-chloroaniline), which was obtained from Sigma-Aldrich. The amount of curative added was calculated to ensure that all free NCO groups in the prepolymer would be completed reacted following the stochiometric ratio. HNT nanotubes, having diameters in the range of 30–70 nm, lengths of 1–3 μm, and surface area of 64 m^2^/g were supplied by Sigma Aldrich.

### Acid treatment of HNTs

The HNTs were dispersed via sonication in distilled water and the sulfuric acid was added slowly to obtain a 3 M solution. The solution was kept under constant stirring for 1 h at 90 °C. The nanotubes were removed from the acid solution via centrifugation and washed with distilled water. The HNTs were dried in a vacuum oven at 120 °C for 12 h and then crushed with a mortar.

### Synthesis of nanocomposite

The HNTs were incorporated into the liquid pre-polymer at a weight fraction of 0.8% and dispersed via sonication prior to the silane termination process. The secondary aminoalkoxy silane was added dropwise at a weight fraction of 0.6% of the pre-polymer weight and stirred at 80 °C for 20 min. An inert atmosphere was maintained during the process to prevent the premature hydrolysis of the siloxane groups. The curative was melted at 110 °C prior adding to pre-polymer, and the mixture was post-cured for 22 h at 120 °C in a metallic mold. Next, the samples were kept at room temperature and with a relative humidity of 50% for 14 days to allow the complete cure.

### Material characterization

The chemical structure of the nanocomposite and neat PU samples were analysed via Fourier Transform Infrared Spectroscopy (FTIR) in a wavenumber range from 600 to 3,600 cm^−1^ using an Agilent Cary 630 spectrometer. The cured polymeric samples used in the FTIR analysis were flat and had dimensions of approximately 80 mm × 20 mm and thickness of 3 mm. Also, were introduced a higher content of aminosilane in the liquid prepolymer (5 wt%) to obtain more clear peaks that evidence the reaction progress. Differential Scanning Calorimetry (DSC) analyses were performed using a TA Instruments DSC Q20, in a temperature range from 30 to 300 °C with a heating rate of 20 K/min. The thermal analysis was conducted based on test method proposed by Frick and Rochman^39^ for thermoplastic PU. The DSC analysis was performed to investigate the influence of the synthesis and process conditions in the resultant crystalline morphology. Scanning Electron Microscopy (SEM) of the recovered samples following the spall tests were recorded using a Tescan Vegal microscope. Energy-dispersive X-ray spectroscopy analysis was conducted together with SEM in order to identify the HNTs in the PU matrix via INCA Energy Dispersive Spectroscopy.

### Spall testing

The experimental parameters and conditions selected for this study are provided in Table 1. To study the effect of HNT reinforcement and silane end-capping on the spall strength of PU, experiments were conducted under conditions that would introduce similar tensile strain rates and comparable shock stresses on both samples. Identical strain rates were achieved between samples by changing the sample thickness of the neat polymer. Fracture toughness comparisons between the neat polymer and HNT-reinforced nanocomposite involved tests involving similar values of peak compressive shock stresses in the samples. Considering the glassy-like response of PU under these strain rate conditions, the spall strength is not assumed to be strongly dependent on the peak compressive shock stress. Similar observations have been made in polymethylmethacrylate^29^.

**Table 1:** Summary of the experimental parameters and conditions.

Target	Flyer	Flyer velocity (m/s)	Target thickness (mm)	$\dot{\varepsilon }_{{\text{u}}}$(10^4^ s^−1^)	${\upsigma }_{{{\text{sp}}}}$(MPa)	$K_{c}$(MPa m^1/2^)	Shock stress^a^ (GPa)
Neat PU 01	1/8" acrylic	436	4.1	2.75	105 ± 2	–	0.78
Neat PU 02	1/8" acrylic	513	8.0	3.16	134 ± 4	3.41 ± 0.16	0.96
PUs HNT 01	1/8" acrylic	493	8.4	2.76	143 ± 3	–	0.93
PUs HNT 02	1/8" acrylic	519	7.5	2.83	149 ± 2	4.13 ± 0.08	0.99
PU HNT 01	1/8" acrylic	450	4.2	2.79	129 ± 3	–	0.93

^a^The peak shock stresses were calculated using the formula $\sigma$ = $\rho$ U_s_u_p._ And, Us by the polynomial approximation Us = 6.486u_p_ − 7.823u_p_ + 3.549u_p_ + 2.703 for up ≤ 0.4 km · s^−1^ for acrylic^40^.
